# Reduced Right Ventricular Function Predicts Long-Term Cardiac Re-Hospitalization after Cardiac Surgery

**DOI:** 10.1371/journal.pone.0132808

**Published:** 2015-07-21

**Authors:** Leela K. Lella, Virna L. Sales, Yulia Goldsmith, Jacqueline Chan, Marina Iskandir, Iosif Gulkarov, Anthony Tortolani, Sorin J. Brener, Terrence J. Sacchi, John F. Heitner

**Affiliations:** 1 Division of Cardiology, New York Methodist Hospital, Brooklyn, NY, 11215, United States of America; 2 Department of Cardiothoracic Surgery, New York Methodist Hospital, Brooklyn, NY, 11215, United States of America; Loyola University Chicago, UNITED STATES

## Abstract

**Background:**

The significance of right ventricular ejection fraction (RVEF), independent of left ventricular ejection fraction (LVEF), following isolated coronary artery bypass grafting (CABG) and valve procedures remains unknown. The aim of this study is to examine the significance of abnormal RVEF by cardiac magnetic resonance (CMR), independent of LVEF in predicting outcomes of patients undergoing isolated CABG and valve surgery.

**Methods:**

From 2007 to 2009, 109 consecutive patients (mean age, 66 years; 38% female) were referred for pre-operative CMR. Abnormal RVEF and LVEF were considered <35% and <45%, respectively. Elective primary procedures include CABG (56%) and valve (44%). Thirty-day outcomes were perioperative complications, length of stay, cardiac re-hospitalizations and early mortaility; long-term (> 30 days) outcomes included, cardiac re-hospitalization, worsening congestive heart failure and mortality. Mean clinical follow up was 14 months.

**Findings:**

Forty-eight patients had reduced RVEF (mean 25%) and 61 patients had normal RVEF (mean 50%) (*p*<0.001). Fifty-four patients had reduced LVEF (mean 30%) and 55 patients had normal LVEF (mean 59%) (*p*<0.001). Patients with reduced RVEF had a higher incidence of long-term cardiac re-hospitalization vs. patients with normal RVEF (31% vs.13%, *p*<0.05). Abnormal RVEF was a predictor for long-term cardiac re-hospitalization (HR 3.01 [CI 1.5-7.9], *p*<0.03). Reduced LVEF did not influence long-term cardiac re-hospitalization.

**Conclusion:**

Abnormal RVEF is a stronger predictor for long-term cardiac re-hospitalization than abnormal LVEF in patients undergoing isolated CABG and valve procedures.

## Introduction

Right ventricular ejection fraction (RVEF) has been demonstrated as an independent predictor of survival in patients with stable heart failure [1]. Patients with abnormal right ventricular (RV) function and co-existing left ventricular (LV) dysfunction have worse outcomes after coronary artery bypass surgery (CABG) [2]. In the current practice guidelines, the evaluation of RVEF is not routinely performed as a pre-operative risk stratification of patients undergoing cardiac surgery [3–5]. However, the significance of RVEF, independent of LVEF, following isolated CABG and valve procedures remains unknown.

Two-dimensional echocardiography is a widely-used technique for calculating RVEF. However, two-dimensional echocardiography provides poor visualization of the RV free wall due to its anterior position beneath the sternum. Moreover, the innate complex multiplanar geometry of the RV makes the calculation of the summation-type volume cumbersome. Cardiac magnetic resonance (CMR) allows accurate measurement of RV volumes, thus overcoming the acoustic limitations inherent in two-dimensional echocardiography. CMR does not make assumptions on the RV geometry and is now considered the gold standard for RVEF assessment [6,7].

The aim of this study is to determine whether abnormal RVEF, independent of LVEF, as assessed by CMR, is a predictor of 30-day and long-term outcomes of patients who undergo elective primary CABG and valve procedures.

## Materials and Methods

### Study Population

We performed a retrospective evaluation of 121 consecutive patients from 2007 to 2009, who were referred to CMR by either a cardiologist or cardiac surgeon for the assessment of LV function, myocardial viability, and valve disease and were scheduled to undergo elective, primary surgical procedures (either CABG or valve surgery). All patients had CMR imaging performed. We excluded 12 patients who required surgery for aortic pathology (ie. aortic aneurysm, dissection) along with concomitant planned CABG and valve procedures. All study patients except in one from the CABG group underwent elective on-pump surgeries. Additional exclusion includes patients with vascular clips or other relevant metallic implants, implanted pacemakers, or defibrillators such as implantable cardioverter defibrillator (ICD) or cardiac resynchronization therapy (CRT). Patient demographics, comorbidities, and medications were obtained from the patient on the day of the study. The study was conducted according to the principles of the 1975 Declaration of Helsinki and was approved by the Institutional Review Board at New York Methodist Hospital. All patients was given a consent form describing the study in detail and was asked to sign this consent form.

### Early and Long-term outcomes

Preoperative, perioperative data, and 30-day as well as long-term outcomes >30 days after surgery were reported based on standard defintions established by The Society of Thoracic Surgeons database [8]. Thirty-day outcomes include length of intensive care unit stay and hospital stay (entire stay from admission to discharge), duration of mechanical ventilation, perioperative complications, and cardiac re-hospitalizations. Perioperative complications include reoperation for bleeding, deep sternal infection, stroke, transient ischemic attack, pacemaker implantation, myocardial infarction, atrial fibrillation and renal insufficiency requiring dialysis (8). Prolonged ventilation, as defined by prolonged intubation time of ≥ 12 hours was established in accordance to our hospital practice. Readmissions to the intensive care unit (ICU) during hospitalization and repeat cardiac hospitalization within 30-days after hospital discharge were both documented. Early (≤ 30-days) mortality included patients who died during hospitalization and patients who died after discharge from the hospital within 30 days of the surgery. Long-term outcomes (> 30-days) included worsening congestive heart failure (CHF), as defined by a worsening in New York Heart Association (NYHA) classification (functional class I to IV, with IV most severe) [8] and cardiac re-hospitalization, defined as hospital admission for acute coronary syndrome, decompensated heart failure or arrhythmia, as assessed by cardiologist chart review and long-term survival, as supplemented from the Social Security Death Index (SSDI) [9]. Data on periperative events and long-term outcomes and survival were available for all patients included in the study cohort.

### Clinical follow-up

Clinical follow-up averaged 14 ± 8.1 months (median 11.8). Every 6 months, follow-up data for patients were obtained after CMR from phone interviews followed by a review of hospital records to confirm cardiac etiology for re-hospitalization. Data on HF severity for worsening NYHA class was determined through a phone interview by a cardiologist. Each of the patients NYHA class at follow-up was compared to their baseline class obtained prior to surgery. Long-term survival follow-up at 100% using the SSDI averaged 59.2± 11.1 months (median 58.0).

### CMR Imaging Protocol and Analysis

**Fig 1:**
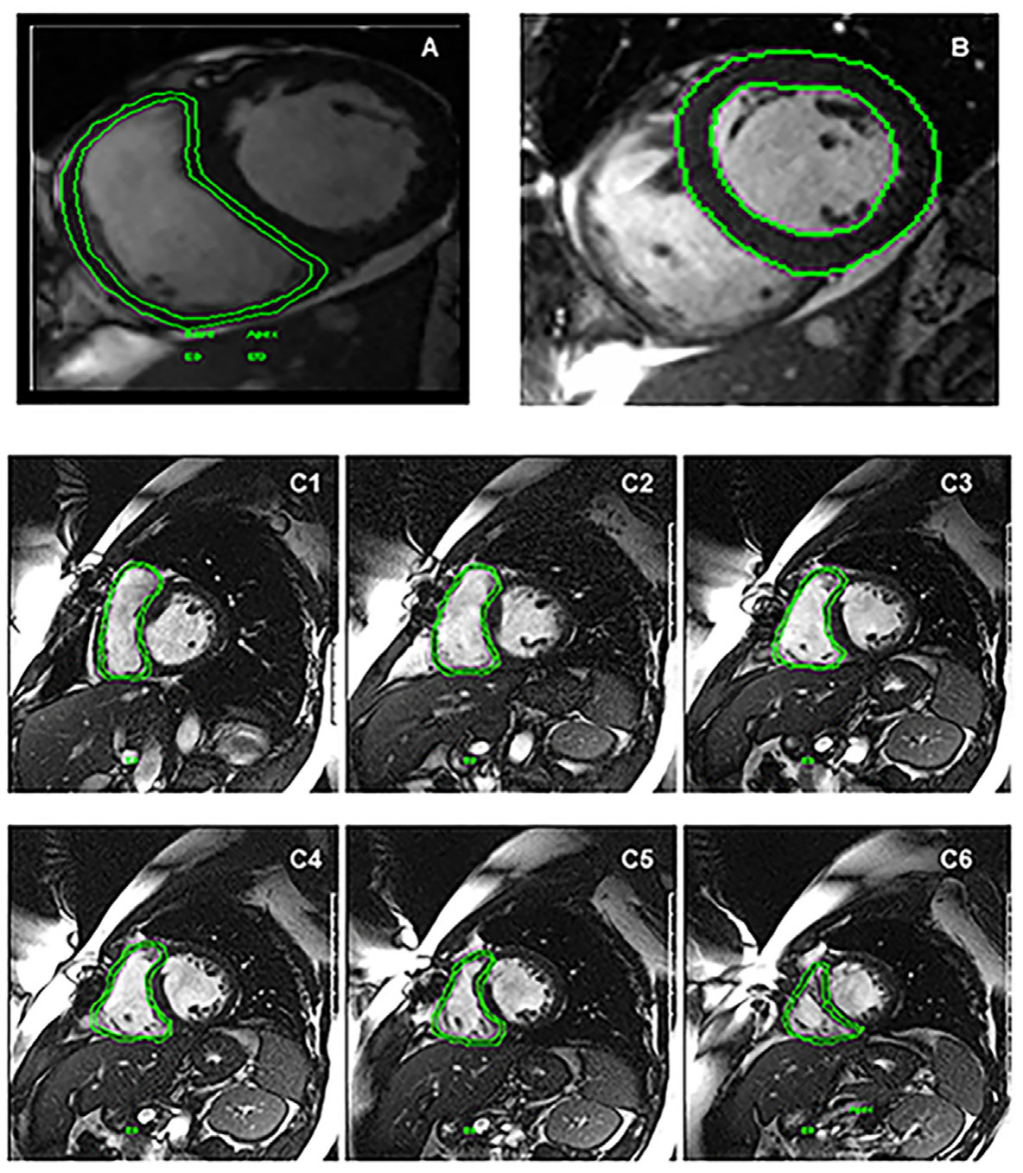
Preoperative CMR. Short axis cine views traced right ventricle (A), left ventricle (B) and right ventricle from base to the apex (C) for ejection fraction measurements. Marked region of interest is demonstrated in green.

All patients had CMR imaging performed according to the study protocol at New York Methodist Hospital. Imaging analysis and reporting were also carried out solely at our institution. CMR was performed using a 1.5-Tesla scanner (Avanto; Siemens, Germany). A steady-state free precession sequence (recovery time, 47.1 ms; echo time, 1.3 ms; flip angle, 90°; bandwidth, 930 Hz/pixel) was used to examine RV anatomy and function. Images were acquired during breath-hold, in short-axis planes (voxel size, 1.4x1.3x6 mm) parallel to the tricuspid valve annulus. Between six and eight short-axis images of the RV were obtained with a slice thicknesses of 6 mm and a gap of 4mm. RV volumes were measured using offline software (Argus; Siemens, Germany). After the identification of the end-systolic and end diastolic phases of the cardiac cycle, semi-automated detection of endocardial borders was optimized with fine manual adjustment, and RV volumes and EF were calculated (Fig 1). Similar methods were used to quantify LV volumes (end-diastolic and end-systolic) and ejection fraction using Argus software on short-axis cine images.

Patients were divided into comparison groups according to the calculated RVEF. Cut-off values for abnormal RVEF and LVEF were < 35% and < 45%, respectively (2). Patients were also divided into two groups according to the type of procedures; CABG (61 patients) vs. valve surgeries (48 patients).

### Statistical Analyses

Demographic variables, comorbidities, medications, blood tests, ventricular function, CMR variables, operative data, 30-day, and long-term outcomes in patients undergoing cardiac surgery were compared between patients with abnormal RVEF vs. normal RVEF. 30-day and long-term outcomes were also compared between patients undergoing CABG vs. valve surgery. Continuous and categorical variables were compared using t-test and Fisher’s exact test, respectively. Multivariable logistic regression models were constructed to identify factors associated with RVEF predictive of poor early (≤ 30-days) and long-term (>30 days) outcomes. Candidate variables include age, sex, CHF, obesity, chronic obstructive pulmonary disease (COPD), tricuspid regurgitation (TR), pulmonary hypertension severity, preoperative RVEF and LVEF, coronary bypass graft surgery (CABG) and valve surgery. Overall survival estimates were obtained by Kaplan-meier method. The groups were compared with a log-rank test. Cox proportional hazards regression was used to discriminate risk factors associated with time of death. All estimates are provided with 95% confidence interval (CI). Candidate variables considered for the multivariable analyses were those detected by univariate models as having a *p* < 0.05 or suggestive trend toward association (*p* = 0.05–0.10) predictive of perioperative and 30-day complications, long-term cardiac re-hospitalization and worsening CHF, and late mortality; retention of variables was set at *p* < 0.05. To discriminate independent risk factors, multivariable modeling was performed with methods of stepwise selection, with RVEF groups and candidate variables all competing for entry into a final model predicting perioperative and 30-day complications, long-term cardiac re-hospitalization, and worsening CHF, and late mortality. Univariate and multivariate analyses were performed in the same manner to identify predictors of late mortality. Probability values were considered significant when *p* < 0.05. All analyses were performed using SPSS 20 (IBM; New York, USA).

## Results

### Study Population

The mean age of the sample was 66 ± 12 years and 38% were women. Baseline characteristics between patients with abnormal and normal RVEF were similar except that normal RVEF group had a higher percentage of female population but lower incidence of COPD. There was no difference in the presence of risk factors for CAD, incidence of CHF and renal failure between the two groups (Table 1).

**Table 1:** Patient Characteristics.

Characteristic	TOTAL	RVEF <35%	RVEF ≥35%	*P-value*
Age (y, mean ± SD)	65.8 ± 12.1	64.2 ± 11.9	67.0 ± 12.3	0.244
Female (n, %)	41 (37.6)	10 (20.8)	31(50.8)	**0.001**
**Coronary artery disease risk factors**				
Diabetes Mellitus (n, %)	45 (41.3)	17 (35.4)	28 (45.9)	0.329
Hypertension (n, %)	91 (84.3)	40 (83.3)	51 (85.0)	1.000
Myocardial infarction (n, %)	16 (14.7)	5 (10.4)	11 (18.0)	0.292
Congestive heart failure (n, %)	23 (37.7)	25 (52.1)	48 (44.0)	0.174
Hyperlipidemia (n, %)	63 (57.8)	30 (62.5)	33 (54.1)	0.437
Family history of CAD (n, %)	21 (19.3)	8 (16.7)	13 (21.3)	0.629
CVA/Transient ischemic attack (n, %)	4 (3.7)	2 (3.3)	2 (4.2)	1.000
Body mass index (kg/m^2^,mean ± SD)	27.5 ± 5.1	28.1 ± 5.9	27.0 ± 2.5	0.279
Obesity (n, %)	27 (24.8)	13 (27.1)	14 (23.0)	0.660
**Past medical history**				
Chronic kidney disease (n, %)	20 (18.3)	10 (20.8)	10 (16.4)	0.622
Chronic obstructive pulmonary disease (n, %)	13 (11.9)	11 (22.9)	2 (3.3)	**0.002**
Atrial fibrillation (n, %)	12 (11.0)	4 (8.3)	8(13.1)	0.544
**Medications on admission**				
β-blocker (n,%)	85 (78.0)	35 (72.9)	50 (82.0)	0.352
Aspirin (n, %)	67 (61.5)	31 (64.6)	36 (59.0)	0.692
ACE-Inhibitors/ Angiotensin receptor blockers (n, %)	72 (66.1)	28 (58.3)	44 (72.1)	0.156
Plavix (n, %)	21 (19.3)	7 (14.6)	14 (23.0)	1.000
Statins (n, %)	61 (56.0)	27 (56.3)	34 (55.7)	1.000
**Blood test**				
Pro-BNP (pg/mL, mean± SD)	3175.9 ± 7045.4	4573.2 ± 8760.4	2081.3 ± 5154.7	**0.069**
Hematocrit (%, mean ± SD)	35.3 ± 5.9	36.1 ± 6.4	34.7 ± 5.4	0.213
Creatinine (mg/dL, mean ± SD)	1.2 ± 0.9	1.1 ± 0.4	1.3 ± 1.1	0.379
Total cholesterol (mg/dL, mean ± SD)	166.0 ± 44.1	159.1 ± 40.2	171.4 ± 46.6	0.149
LDL (mg/dl, mean ± SD)	97.3 ± 37.5	92.7 ± 35.7	101.0 ± 38.9	0.254
HDL (mg/dl, mean ± SD)	47.5 ± 15.9	44.2 ± 13.5	50.1 ± 17.2	0.056
Triglycerides mg/dl, mean ± SD)	120.3 ± 64.8	119.2 ± 64.3	121.2 ± 65.8	0.861
**Right ventricle**				
RVEF (% mean± SD)	39.2 ± 15.1	25.4 ± 7.6	50.0 ± 9.5	**0.000**
RV abnormal contraction (n, %)	36 (33.0)	17 (35.4)	19 (31.1)	0.685
RV dilation (n, %)	22 (20.2)	9 (18.8)	13 (21.3)	0.813
Tricuspid regurgitation (mean ± SD)	0.9 ± 1.0	0.9 ± 0.9	0.9 ± 1.0	0.818
Tricuspid regurgitation 2+ (n,%)	27 (24.8)	13 (27.1)	14 (23.0)	0.660
Pulmonary Hypertension (n, %)	42 (38.5)	23 (47.9)	19 (31.1)	0.079
Mild [mmHg, RVSP 40–45 or mPAP 26–40] (n, %)	18 (16.5)	11 (22.9)	7 (11.5)	0.126
Moderate[mmHg, RVSP 46–54 or mPAP 41–59] (n,%)	19 (17.4)	8 (16.7)	11 (18.0)	1.000
Severe [mmHg, RVSP ≥55 or mPAP ≥ 60] (n, %)	5 (4.6)	3 (6.3)	2 (3.3)	0.653
**Left ventricle**				
LVEF (%, mean ± SD)	44.7 ± 17.5	39.0 ± 16.8	49.1 ± 17.0	**0.002**
LVEF < 45% (n, %)	54 (49.5)	29 (60.4)	25 (41.0)	0.055
LV Dysfunction (n,%)	41 (37.6)	19 (39.6)	22 (36.1)	0.842
LV Hypertrophy (n, %)	40 (36.7)	20 (41.7)	20 (32.8)	0.424
**Cardiac MRI measurements**				
Scar (%, mean ± SD)	3.1 ± 5.1	4.1 ± 5.7	2.3 ± 4.6	0.083
LV mass (g, mean± SD)	104.5 ± 91.3	109.4 ± 47.5	100.5 ± 114.9	0.619
**Procedures**				
Coronary artery bypass graft only (n, %)	61 (56.0)	28(58.3)	33 (54.1)	0.701
Valve only (n, %)	48(44.0)	20 (41.7)	28 (45.9)	0.701
Mitral valve surgery (n, %)	27 (24.8)	12(25.0)	15(24.6)	1.000
Tricuspid valve surgery (n, %)	2(1.80)	0 (0.0)	2 (3.3)	0.503
Aortic valve surgery (n, %)	28 (25.7)	15 (31.3)	13 (21.3)	0.274
Concomitant procedures (Aortic/AF ablation (n, %)	3 (2.8)	0 (0.0)	3 (4.9)	0.254
Double valves ± Concomitant procedures (n, %)	9 (8.3)	4 (8.3)	5 (8.2)	1.000
Cardiopulmonary bypass time (minutes, median[IQR])	70.0 (191.0)	72.5 (149.0)	69.0 (191.0)	0.962
Cross clamp time (minutes, median [IQR])	41.0 (115.0)	44.5 (104.0)	39.0 (114.0)	0.765
Cardioplegia- Antegrade (n, %)	16 (14.7)	9(18.8)	7(11.5)	0.414
- Antegrade-Retrograde (n, %)	93 (85.3)	39(81.5)	54(88.5)	

Cerebral vascular accident, CVA, Pro-BNP, pro-brain natriuretic peptide; LDL, low-density lipoprotein; HDL, high density lipoprotein; RVEF, right ventricular ejection fraction; RVSP, right ventricular systolic pressure; mPAP, mean pulmonary artery pressure; LVEF, left ventricular ejection fraction. Measurements of RV and LV volumes, mass, regional wall motion abnormalities, and function were obtained by CMR; TR and PAP by echocardiography.

Forty-eight patients had reduced RVEF (mean 25%). Sixty-one patients had normal RVEF (mean 50%). Fifty-four patients had reduced LVEF (mean 30%) and 55 patients had a normal LVEF (mean 59%). There was a significant difference in mean LVEF among patients with abnormal RVEF and normal RVEF (LVEF 39% vs 49%, respectively, *p* = 0.002).

There were no statistically signficant differences in type of surgery performed and in cardioplegia used among RVEF groups. In patients undergoing isolated CABG procedures, median perfusion and cross clamp times were 70 minutes (range 30–191) and 40 minutes (range 18–129), respectively, with one case of off-pump surgery (data not shown).

### Outcomes

#### Perioperative events and early outcomes (≤ 30-days)

One hospital death occurred in a patient who had valve surgery. No 30-day repeat cardiac hospitalization was observed. There were no differences in the duration of mechanical intubation (*p* = 0.5), length of ICU (*p* = 0.5) and hospital stays (*p* = 0.2) and early complications (*p* = 0.8) between patients with abnormal RVEF compared to normal RVEF (Table 2). When patients were stratified by type of surgery, there were no statistically significant differences in the incidence of prolonged ventilation (≥ 12 hours), perioperative complications, length of ICU and hospital stays, and 30-day mortality between RVEF groups for patients undergoing CABG procedures. These findings were similar in valve surgical patients (Table 2). Of those patients undergoing valve procedures (vs.CABG), prolonged ventilation (≥ 12 hours) and longer hospital stay were most common (81% vs. 54%, *p* = 0.004; 18 vs.14 days, *p* = 0.041, respectively) (Table 3).

**Table 2:** Analysis of early (≤ 30-d) and long-term (> 30-d) outcomes associated with RVEF <35% and RVEF ≥ 35%.

Variables	TOTAL	RVEF <35%	RVEF ≥35%	*P*-value
	n = 109	n = 48	n = 61	
**Early outcomes (≤ 30 d)**				
Creatinine (mg/dL, mean ± SD)	1.2 ± 1.2	1.1 ± 0.6	1.4 ± 1.4	0.215
Ventilation time (hours, median [IQR])	15.0 (2660.0)	14.0 (2660.0)	16.0 (812.0)	0.798
Prolonged Ventilation (≥12 hours, n, %)	72 (66.1)	30 (62.5)	42 (68.9)	0.544
ICU (days, median [IQR])	7 (40.0)	8 (35.0)	7 (40.0)	0.507
Length of stay (days, mean ± SD)	15.9 ± 10.5	17.4 ± 11.6	14.7± 9.5	0.175
Elevated LFT (n, %)	4 (3.7)	1 (2.1)	3 (4.9)	0.629
Complications (n, %)	34 (31.2)	14 (29.2)	20 (32.8)	0.835
Discharge and 30-d Mortality (n, %)	1 (0.9)	0 (0.0)	1 (1.6)	1.000
Re-hospitalization for cardiac cause (≤30-d, n, %)	0 (0.0)	0 (0.0)	0 (0.0)	—
**Long-term outcomes (>30-d)**				
Worsening congestive heart failure (>30-d, n, %)	40 (36.7)	21 (43.8)	19 (31.1)	0.230
Re-hospitalization for cardiac cause (> 30-d, n, %)	23 (21.1)	15 (31.3)	8 (13.1)	0.032
All-cause mortality (>30-d, n, %)	16 (14.7)	5 (10.4)	11(18.0)	0.292
**Outcomes in CABG or Valve surgery alone**	61 (56.0)	28 (58.3)	33 (54.1)	0.701
Coronary artery bypass graft alone (n, %)				
Prolonged ventilation (≥ 12 hours, n, %)	33 (54.1)	14 (50.0)	19 (57.6)	0.612
ICU (days, median [IQR])	7 (30.0)	8 (25.0)	7 (30.0)	0.507
Early complications (n, %)	17 (27.9)	8 (28.6)	9 (27.3)	1.000
In-hospital and 30-d Mortalities (n, %)	0 (0.0)	0 (0.0)	0 (0.0)	—
Length of stay (days, mean ± SD)	16.0 ± 11.8	15.8 ± 9.7	12.5± 6.7	0.175
Worsening congestive heart failure (>30-d, n, %)	21 (34.4)	12 (42.9)	9 (27.3)	0.281
Re-hospitalization for cardiac cause (>30-d, n, %)	10 (16.4)	6 (21.4)	4 (12.1)	0.490
Bleeding requiring reoperation (> 30-d, n, %)	0 (0.0)	0 (0.0)	0 (0.0)	—
All-cause long-term mortality (>30-d, n, %)	11(18.0)	4 (14.3)	7 (21.2)	0.526
Valve surgery alone (n, %)	48 (44.0)	20 (41.7)	28 (45.9)	0.701
Prolonged ventilation (≥ 12 hours, n, %)	39 (81.30)	16 (80.0)	23 (82.1)	1.000
ICU (days, median [IQR])	8 (40.0)	8 (35.0)	8 (39.0)	0.507
Early complications (n, %)	17 (35.4)	6 (30.0)	11 (39.3)	0.555
In-hospital and 30-d Mortalities (n, %)	1 (2.1)	0 (0.0)	1 (3.6)	1.000
Length of stay (days, mean ± SD)	18.2 ± 12.5	19.6 ± 13.8	17.1± 11.7	0.175
Worsening congestive heart failure (> 30-d, n, %)	19 (39.6)	9 (45.0)	10 (35.7)	0.561
Re-hospitalization for cardiac cause (> 30-d, n,%)	13 (27.1)	9 (45.0)	4 (27.10)	0.025
Bleeding requiring reoperation (> 30-d, n, %)	0 (0.0)	0 (0.0)	0 (0.0)	1.000
All-cause long-term mortality (>30-d, n, %)	5 (10.4)	1(5.0)	4(14.3)	0.385

ICU, intensive care unit; IQR, interquartile range; LFT, liver function test; postoperative complications (i.e. reoperations for bleeding, early valve reoperations, deep sternal infection, early stroke, early transient ischemic attack, pacemaker implantation, myocardial infarction, atrial fibrillation and renal insufficiency requiring dialysis).

**Table 3:** Analysis of early (≤ 30-d) and long-term (> 30-d) outcomes associated with primary CABG and valve procedures.

CABG vs. VALVE	TOTAL	CABG	VALVE	*P*-
	n = 109	n = 50	n = 59	value
		(45.9%)	(54.1%)	
Prolonged ventilation (≥ 12 hours, n, %)	72 (66.1)	33 (54.1)	39 (81.3)	0.004
Early complications within 30-d (n, %)	34 (31.2)	17 (27.9)	17 (35.4)	0.413
Length of stay (days, mean ± SD)	15.9 ± 10.5	14.0 ± 8.3	18.2± 12.5	0.041
Worsening congestive heart failure (> 30-d, n, %)	40 (36.7)	21 (34.4)	19 (39.6)	0.689
Re-hospitalization for cardiac cause (> 30-d, n, %)	23 (21.1)	10 (16.4)	13 (27.1)	0.237
All-cause long-term mortality (>30-d, n %)	16 (14.7)	11(18.0)	5 (10.4)	0.292

Early complications within 30-d include reoperations for bleeding, early valve reoperations, deep sternal infection, early stroke, early transient ischemic attack, pacemaker implantation, myocardial infarction, atrial fibrillation, and renal insufficiency requiring dialysis.

#### Long-term outcomes (> 30-days)

The incidence rate in worsening CHF appears to be higher in abnormal RVEF compared with normal RVEF, but the increase is not statistically signficant (Table 2). There were more cardiac re-hospitalizations in the abnormal RVEF group when compared to the normal RVEF group (31% vs. 13%, *p* = 0.032). However, of those 23 patients with long-term cardiac re-hospitalizations, risk factors associated with RV dysfunction were low in abnormal group (i.e. COPD 17%, TR2+ 26%, and pulmonary hypertension 39%). TR was graded as 0 for no regurgigation, 1+ for mild regurgitation, 2+ for moderate regurgitation, 3+ for moderately severe regurgitation, and 4+ for severe regurgitation [8,10]. When patients were stratified by type of surgery, there were no statistically significant differences in long-term worsening CHF or cardiac re-hospitalization between RVEF groups for CABG patients. Among RVEF groups undergoing valve surgery, the percentage of patients with late worsening CHF was similar, however, a greater number of patients with abnormal RVEF had late cardiac re-hospitalization (45% vs. 27%, *p* = 0.025). There were no statistically signficant differences in the incidence of long-term worsening CHF and cardiac re-hospitalization among CABG and valve surgeries (Table 3).

#### Risk factors for worse early (≤ 30 days) and long-term (≥ 30 days) outcomes

Multivariate analysis (including LVEF) revealed that abnormal RVEF conferred an independent and significant risk for cardiac re-hospitalization at long-term with a HR = 3.01 (CI: 1.1–7.9, *p* = 0.032) (Table 4). LVEF did not influence cardiac re-hospitalization. Neither RVEF nor LVEF were predictors of poor early outcomes and death (Table 4). Risk factors associated with RV dysfunction included incidence of CHF, obesity, COPD and pulmonary hypertension did not affect early and long-term outcomes (data not shown). However, higher incidence of significant TR and advanced age were predictive of worse survival (Table 4). In the early postoperative phase (≤ 30 days), valve surgery increased risk for higher number of complications (Table 3), but not in the long-term phase (> 30 days) (Table 4). However, the results of the multivariate analysis should be interpreted with caution, given the relatively small number of events in this patient group as reflected by large confidence intervals.

**Table 4:** Risk factors associated with poor early (≤30-d) and long-term (>30-d) outcomes.

Variable			
	Hazard Ratio	95% CI	*P*-Value
**Early (≤ 30-d) outcomes**			
Older age	1.014	0.978–1.052	0.454
RVEF<35%	0.911	0.367–2.264	0.841
LVEF<45%	1.242	0.492–3.138	0.647
Valve surgery	2.930	1.060–8.102	**0.038**
**Long-term Rehospitalization for cardiac cause**			
RVEF<35%	3.011	1.151–7.879	**0.025**
LVEF<45%	1.172	0.203–6.772	0.860
Valve surgery	1.753	0.307–9.999	0.528
Tricuspid Regurgitation 2+	1.689	0.195–14.630	0.634
**Late mortality**			
Older age	1.064	1.001–1.132	**0.048**
Tricuspid Regurgitation 2+	2.968	1.039–8.478	**0.042**

RVEF, right ventricular ejection fraction; LVEF, left ventricular ejection fraction; CI, confidence interval.

#### Long-term survival

**Fig 2:**
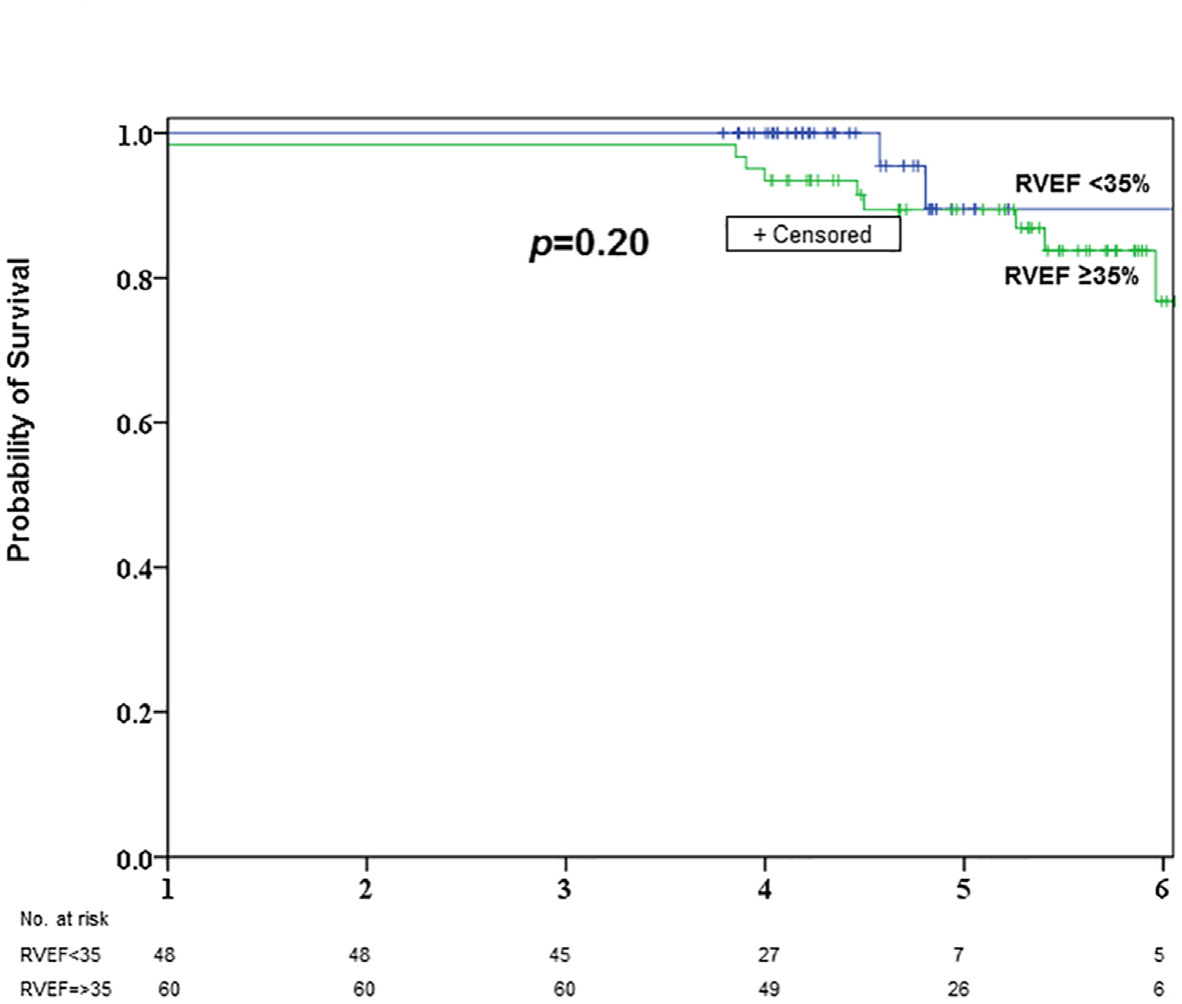
Kaplan-meier survival curve comparing patients with RVEF < 35% (n = 48) and RVEF≥ 35% (n = 61) undergoing CABG or valve procedures. The top curve corresponds to patients with RVEF <35% and the bottom curve to patients with RVEF≥ 35%. The number below each year indicates the number of patients at risk.

Overall unadjusted estimated survival at 1,3, and 5 years was 99%, 99%, and 91%, respectively. Survival in the abnormal RVEF group was 100%, 100%, and 92%, respectively, and in the normal RVEF group it was 98%, 98%, and 90%, respectively. Survival was similar among RVEF groups (*p* = 0.201) (Fig 2). The type of surgery did not affect long-term survival (Tables 3 and 4).

## Discussion

In this study, abnormal RVEF, measured by CMR, was an independent risk factor for long-term cardiac re-hospitalizations, and a better predictor of cardiac re-hospitalization compared to abnormal LVEF. In addition, patients who underwent valve surgery with an abnormal RVEF had an increased incidence of late repeat cardiac hospitalizations.

The importance of LV function on outcomes after CABG has been well established in the literature [11]. In recent years, attention has been focused on the RV function and its association with morbidity and mortality. The RV is affected by changes in afterload, preload, and contractility [12,13]. In surgical patients, some consideration of RV function is warranted as many surgically relevant disease states may potentially alter such determinants of RV function [14,15].

In the Should We Emergently Revascularize Occluded Coronaries for Cardiogenic Shock (SHOCK) trial, patients presenting with right ventricular shock, despite younger age and with single vessel disease, had higher mortality than those patients with left ventricular shock [16]. RV dysfunction was predictive of worse survival in patients with pulmonary hypertension [17] and in symptomatic heart failure [18]. In our study, nearly half of patients with abnormal RVEF had pulmonary hypertension than those with preserved RVEF (*p* = 0.079). In a study of 147 patients, Adhyapak and colleagues reported that rapid progression occurred more in patients with abnormal RVEF than those with preserved RVEF, irrespective of the co-existing pulmonary hypertension in both groups [18]. Our data showed that abnormal RVEF led to long-term cardiac re-hospitalizations. This may be partly attributable to the failure of the of right ventricle to maintain flow required to have adequate LV preload, thus leading to progressive decompensation [18].

Reichert and associates [19] observed in 52 post-surgical patients with hypotension that despite of inotropic support, patients with RV dysfunction had a high in-hospital mortality (82%). Mortality was significantly less (30%-40%) in patients with severe LV dysfunction and normal or mildly impaired RV function [19]. Transient decline in right ventricular systolic function after cardiopulmonary bypass has been previously described [14], however, the magnitude of the long-term impact of post-RV dysfunction, if any, is unknown [20]. We cannot reliably predict which patients will have transient RV dysfunction and which patients will have persistent RV dysfunction [21].

Additional studies have shown that clinically silent severe RV enlargement may occur in asymptomatic patiens with normal right ventricular pressure and no pulmonary hypertension and mitral disease [22,23]. Messika-Zeitoun and associates [23] initially found only 27% with severe right-sided chamber enlargement and the rest had normal right-sided chamber dimension. By five years, severe right-sided chamber enlargement also developed in patients who initially did not have any chamber enlargement (39% no enlargement vs. 87% with enlargement, *p* < 0.01). Among these patients, the 15-year probability of HF, atrial fibrillation, cardiac surgery, or death was 69% [22,23].

Our findings demonstrate that the higher incidence of TR2+ is associated with overall worse long-term survival. It is possible that the presence of signficant TR may mask the decreased contractility of the RV, leading to an underestimation of patients who actually have impaired RV function. Clinically silent functional and structural cardiac changes as consequence of TR may have occurred even in asymptomatic cardiac surgical candidates with “normal” RVEF. Earlier work showed that TR severity correlates with poor surival in patients with apparently well-adapted RV function but early reduced contractile reserve [22]. Future CMR studies assessing asymptomatic patients with TR2+ but normal RV size and good RV function by the time of surgical referral may prove informative.

Maslow and colleagues reported that low RVEF (≤ 35%) was associated with worse outcomes than RVEF (> 35%), as measured on echocardiogram, in patients with severe LV dysfunction undergoing elective CABG (n = 41) [2]. In our study of 109 patients, mean LVEF was 45%; patients with low RVEF had mean LVEF of 39%, as measured by CMR. Evaluation of RVEF in the postoperative setting and by echocardiogram is technically challenging [24,25]. Recently, CMR has emerged as a highly reproducible and accurate modality in evaluating RVEF in surgical patients [25–28]. Although our patient population is small, it is one of the larger studies, which has demonstrated the prognostic value of RVEF using CMR following cardiac surgery.

In our series of patients undergoing primary CABG, mean pre-operative RVEF was 25%. The incidence of low RVEF (< 35%) was 58%. Majority of this patient cohort had impaired LV function (LVEF <45% in 57%). Our high-risk selected patients who were undergoing isolated CABG procedures (mean age 67 years, female gender in 60%, diabetes mellitus in 46% and hypertension in 85%) in the current era benefit from improved decreased short-term complications, such as no operative mortality, no cardiac re-hospitalizations, no stroke, renal failure requiring dialysis, reoperation for bleeding, or sternal wound infection, as demonstrated by improved medication adherence: statins in 79%, aspirin in 67%, beta-blockers in 79% and angiotensin-converting enzyme inhibitors in 67%; operative performance and postoperative critical care. Our data demonstrated that LVEF does not appear to increase risk of early complications and long-term mortality in this patient cohort. This finding is consistent with contemporary studies of isolated CABG [29–32]. Two large studies, one containing more than 2000 patients, confirmed that LVEF ≤ 30% was not an independent risk factor of worse early outcomes [29,30]. Nardi and colleagues examined a cohort of 300 patients and observed that LVEF ≤ 20% did not affect overall survival (4–16 years) [31]. In a recent study of over 6000 patients, Yoo and colleagues found that severe LV dysfunction was not associated with reduced long-term survival (2–8 years) [32]. However, other contemporary studies do not support these findings [33,34]. It is important to acknowledge that LVEF is certainly an important prognostic factor in less selected populations.

### Study Limitations

This is a single-institution, nonrandomized study. As in many observational studies, we acknowledge that indication bias is a particular problem in our study. However, we attempted to minimize this by use of restriction and high-quality data. Enrollment in our study was restricted to patients referred for CMR study by the cardiologists and cardiac surgeons. Evaluation of RVEF was performed as a pre-operative risk assessment in those patients. Thus, both groups were similar with respect to most characteristics, comorbid conditions and type of procedures. The quality of data was complete and accurate as obtained from electronic and administrative databases, as well as phone interviews by a cardiologist. Due to our limited patient cohort, we were unable to perform detailed subset analysis using RVEF to predict outcomes on patients with preoperative pulmonary hypertension undergoing cardiac surgery, in order to draw a meaningful conclusion. As in all observational studies compounded by our limited patient cohort, associations could reflect confounding by unmeasured or poorly measured confounders. It is also possible that our results might not be generalizable to patients undergoing straightforward CABG or valve procedures. Future well-powered randomized studyes can adequately these problems.

## Conclusion

In carefully selected patients with preoperative low RVEF and mild-to- moderate LV impairement detected on CMR, isolated CABG or valve procedures may be a safe option, yielding favorable short-term outcomes. Abnormal RVEF emerged as a stronger predictor for long-term cardiac re-hospitalization than abnormal LVEF in our selected patients undergoing elective primary CABG and valve operations. RVEF, measured by right ventricular systolic function on CMR, could serve as a useful parameter in the pre-operative risk stratification of cardiac patients.
